# A Multifunctional Molecular Probe for Detecting Hg^2+^ and Ag^+^ Based on Ion-Mediated Base Mismatch

**DOI:** 10.3390/s18103280

**Published:** 2018-09-29

**Authors:** Luhui Wang, Yingying Zhang, Yafei Dong

**Affiliations:** 1 Department of Life Science, Shaanxi Normal University, Xi’an 710119, China; 2 Department of Computer Science, Shaanxi Normal University, Xi’an 710119, China; zhangyingying@snnu.edu.cn; 3 National Engineering Laboratory for Resource Developing of Endangered Chinese Crude Drugs in Northwest of China, Xi’an 710119, China

**Keywords:** biosensor, logic gate, multifunction molecular probe, fluorescence signal, actual sample detection

## Abstract

In this paper, a multifunctional biosensing platform for sensitively detecting Hg^2+^ and Ag^+^, based on ion-mediated base mismatch, fluorescent labeling, and strand displacement, is introduced. The sensor can also be used as an OR logic gate, the multifunctional design of sensors is realized. Firstly, orthogonal experiments with three factors and three levels were carried out on the designed sensor, and preliminary optimization of conditions was performed for subsequent experiments. Next, the designed sensor was tested the specificity and target selectivity under the optimized conditions, and the application to actual environmental samples further verified the feasibility. Generally, this is a convenient, fast, stable, and low-cost method that provides a variety of ideas and an experimental basis for subsequent research.

## 1. Introduction

With the further development of science and the deepening of research in various disciplines, we have found that some problems cannot be solved perfectly though a single subject, so the concept and research methods of interdisciplinary subjects have emerged. As a typical representative of interdisciplinary research, biocomputing is an emerging computing technology that combines computer science, biology, mathematics, and chemistry. Compared with traditional computing, biocomputing has the advantages of fast parallel processing and high molecular parallelism and has attracted wide attention and rapid development [1]. In recent years, biocomputing has become the focus and hotspot of cutting-edge scientific research, with its superior information storage capacity and low loss, and certain progress and development have been made in theoretical research and application [2,3]. Biological computing has evolved from experiment to practice, single to multiple, and simple to complex [4,5,6]. Constructing simple molecular logic gates [7] and interconnecting the relationships between these logic gates and a more complex logic loop is the basis for building biocomputers. Nowadays, many scientists also use logic gates as biosensors for simultaneous detection, in applications such as environmental monitoring, forensic identification, medical diagnosis, and food testing, using certain characteristics of DNA strands [8,9,10,11,12]. The method not only satisfies the purpose of constructing various logic gates [13] but also overcomes the defects in current detection methods and realize the functional connection between molecular logic gates and biosensor functions. Therefore, it is particularly important to study and discuss this kind of multifunctional biosensor and propose new ideas in this research field.

The progress of society and the development of science and technology have made the use of various ions more and more extensive [14,15,16]. Heavy metal ions can enter the human body through the food chain, causing various hazards to human health. For example, once Hg^2+^ enters the human body, it cannot be excreted by human metabolism [17] and accumulates in the human central nervous system, digestive system, and the kidneys [18,19]. This leads directly to heart, liver, and thyroid diseases, and causes nervous system disorders, chronic Hg^2+^ poisoning, and even the formation of malignant tumors [20]. Although Ag^+^ is less harmful than Hg^2+^, a large amount of Ag^+^ is discharged into the environment in industrial waste every year, as a result of its wide use in photography and pharmaceutical industries [21]. Ag^+^ is highly toxic to bacteria, viruses, algae, and fungi [22]. Once it reaches high concentration, Ag^+^ will exert negative effects, causing environmental pollution (such as water pollution) and endangering human health, as the presence of Ag^+^ denatures the body’s proteins and various enzymes. When more than 0.8 g of Ag^+^ enters the body, it will cause silver spots on the skin [23]. Since Ag^+^ is highly oxidizing, it can cause symptoms such as edema of the internal organs once it enters the human body, causing death in severe cases. Similar to Hg^2+^, the human body does not have a mechanism to effectively discharge Ag^+^. Once Ag^+^ is ingested, it mainly accumulates in the liver and bones, replacing the essential metal ions, such as Ca^2+^ and Zn^2+^, in the hydroxyapatite in the bone [24]. The conventional methods for detecting heavy metal ions include cold vapor atomic fluorescence spectrometry [25], cold atomic absorption spectrometry [26], inductively coupled plasma mass spectrometry [27], and X-ray spectrometry [28]. But these detection methods often require expensive and sophisticated instruments and a large amount of time. Therefore, the development of convenient, fast, and cost-effective methods for detecting heavy metal ions in the environment has become one of the hot issues in current research.

In recent years, researchers have developed many biosensing platforms for detecting heavy metal ions. Since Miyake et al. demonstrated that Hg^2+^ can bind and stabilize two thymine bases by forming a T–Hg^2+^–T binding mismatch, and Ag^+^ is able to specifically interact with the C–Ag^+^–C mismatch in a DNA duplex [29,30,31], many researchers have applied these properties to the detection of Hg^2+^ and Ag^+^ in water and the environment. On this basis, many new methods for detecting Hg^2+^ and Ag^+^ have been proposed, such as the G-quadruplexes detection method [32,33], the fluorescence detection method [34,35], the electrochemical detection method [11,36,37,38,39], the DNA-modified gold nanoparticles detection method [11,16,21,40], the colorimetry detection method [41,42] and the probe microscope detection method [43]. These methods have the advantages of low cost, high efficiency, time efficiency, simple operation, high sensitivity, and specificity, and they completely overcome the disadvantages of traditional detection methods [44].

Motivated by the above arguments, we designed a single-molecule multifunctional DNA logic model which can be used to detect specific metal ions on the basis of strand replacement, ion-mediated base mismatch and fluorescence labeling. In the field of ion detection, we used Hg^2+^ and Ag^+^ as the objects to be detected, and the presence of Hg^2+^ or Ag^+^ was detected by changes in fluorescence intensity within the system. We constructed an OR logic gate by taking Hg^2+^ and Ag^+^ as input and changes in fluorescence intensity as output. In addition, in the orthogonal experiment, condition optimization and performance testing were carried out to verify the feasibility and practical significance of the design.

## 2. Experimental Section

### 2.1. Materials

All DNA was purchased from Sangon Biotechnology Co., Ltd. (Shanghai, China) and purified by PAGE and ULTRAPAGE. DNA sequences are listed in Table 1. All DNA strands were dissolved in ultrapure water as stock solutions (10 μM). Cl_2_HgO_8_·3H_2_O, AgNO_3_ and other reagents were purchased from Xi’an JingBo Bio-Technique Co. (Xi’an, China). The stock solutions of Hg^2+^ and Ag^+^ were prepared by dissolving the desired amount of the materials in ultrapure water, and were diluted to 10 mM, 1 mM, 50 μM and 1 μM.

### 2.2. Fluorescent Signal Detection

In this study, we chose to label substrates with the fluorophore FAM and the quencher BHQ. The fluorescence results were obtained for FAM at 492 nm excitation and 518 nm emission using a fluorescence-scanning spectrometer (EnSpire ELISA; PerkinElmer, Waltham, MA, USA).

## 3. Principles

**Figure 1:**
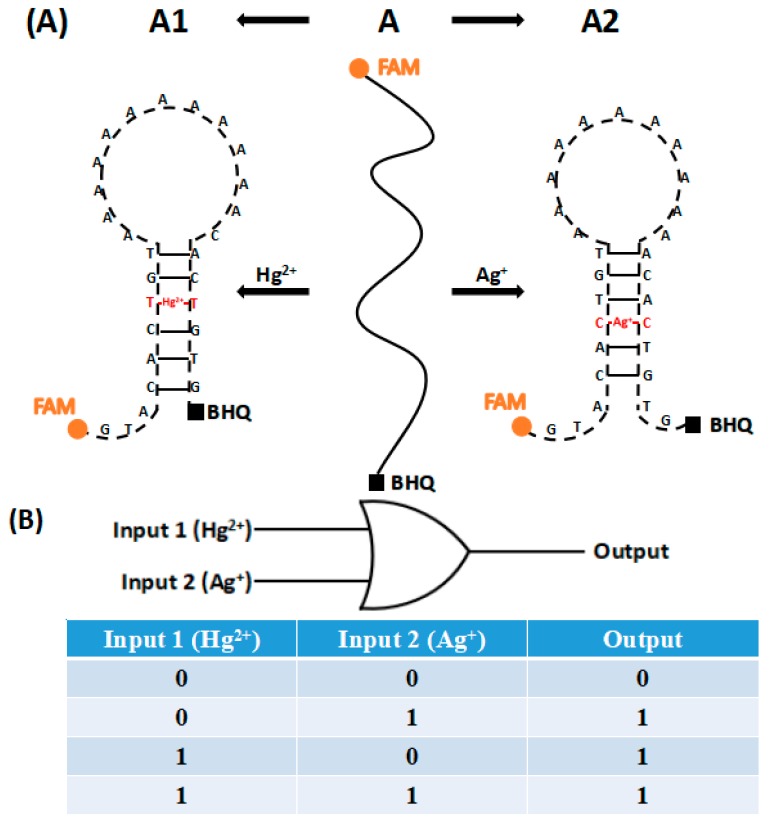
Schematic diagram of the principle of ion detection (**A**). The OR logic gate and the true value table (**B**).

As shown in Figure 1A, we designed a probe DNA (A) with BHQ quenching group and FAM fluorescent group labeling at its 3′ and 5′ ends, respectively. In the absence of Hg^2+^ or Ag^+^, the A strand is in the state of dispersion and spin, the BHQ quenching group of the 3′ end is far away from the fluorescent group of the 5′ end, and the fluorescence intensity of the system is high. The presence of target Hg^2+^ mediates the formation of a T–Hg^2+^–T mismatch, which makes the A strand form a hairpin structure in the A1 form. Similarly, the presence of target Ag^+^ mediates the formation of a C–Ag^+^–C mismatch, which makes the A strand form a hairpin structure in the A2 form. When one of the two targets is present, the structure of A is transformed from single-stranded to the hairpin A1 or A2, which has partially complementary double strands. At this time, the quenching group BHQ of the 3′ end of the strand is close to the fluorescent group FAM of the 5′ end and quenches the fluorescence by fluorescence co-energy transfer, and the fluorescence intensity of the system is greatly reduced. Therefore, changes in the conformation of the A strand and the fluorescence intensity in the system can effectively detect whether Hg^2+^ or Ag^+^ are present in the object to be measured. The sensor has also built a simple OR logic gate, the true value table of which is shown in Figure 1B.

## 4. Results and Discussion

### 4.1. Orthonormal Preliminary Optimization

In the initial stage of the experiment, we designed an orthogonal experiment of three factors at three levels, preliminarily verified the feasibility of the experiment, and obtained the general optimization conditions. The factor levels are shown in Table 2.

There are two important parameters in the orthogonal experimental analysis: *K_ji_* and *R_j_*. *K_ji_* is defined as the sum of the evaluation indexes of all levels (*i*, *i* = 1, 2, 3) in each factor (*j*, *j* = A, B, C) and $\bar{Kji}$ (mean value of *K_ji_*) is used to determine the optimal level and the optimal combination of factors. *R_j_* is defined as the range between the maximum and minimum value of $\bar{Kji}$ and is used for evaluating the importance of the factors [45,46,47,48].

The orthogonal experiment was carried out three times to reduce error. With all the factor settings, considering the orthogonal experimental results (Table 3) and minimizing cost and time, the sixth set of experimental conditions were finally chosen. It is worth mentioning that in order to distinguish better, the normal font data in the table is the experimental results for Hg^2+^, while the italic data is the experimental results for Ag^+^. The reaction time was 30 min, the concentration of the A strand was 1 μM, and the concentration of Ag^+^ or Hg^2+^ was 500 nM, which served as a constant for subsequent optimization of distribution. Furthermore, we can draw a preliminary conclusion based on the R value that the concentration of the A strand had the greatest effect on the experiment, and the reaction time had the least effect on the experiment.

### 4.2. Optimization of Reaction Conditions

In order to obtain optimal reaction conditions, the pH value, concentration of the A strand, reaction temperature and reaction time were optimized. Each experiment was repeated five times, and all the error bars in the graphs were the SD.

**Figure 2:**
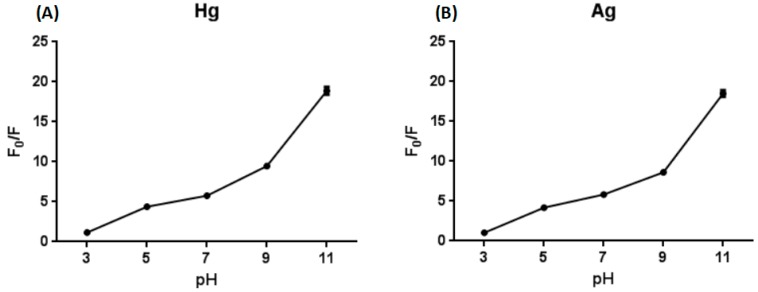
Optimization of pH in the presence of Hg^2+^ (**A**). Optimization of pH in the presence of Ag^+^ (**B**).

If we want to use the designed biosensor for detecting the concentration of ions in actual water samples, pH is an interference factor which must be taken seriously, so the pH value of the reaction system was optimized. It was found that the result was not good under acidic conditions, but the fluorescence value greatly improved under alkaline conditions, and the sensor could function in the pH range of 5–9 (Figure 2). The reason for such a result is that fluorescein may form a spiro ring under acidic conditions, the degree of conjugation is insufficient and the luminescence is not in the visible region. Under alkaline conditions, a highly conjugated structure is formed, and the skeleton is rigid, so the quantum yield is high and the luminescence is strong. On the one hand, the designed sensor can function to detect Hg^2+^ or Ag^+^ under neutral or weak acid and weak alkaline conditions (pH 5–9). On the other hand, the characteristics of the sensor—that the fluorescence intensity under acidic conditions is extremely low and under alkaline conditions is extremely high—can be used to preliminarily determine whether the water body is strongly acidic or alkaline.

**Figure 3:**
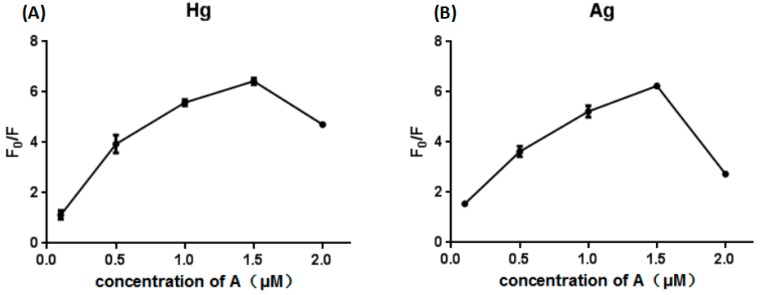
Optimization of concentration of A stand in the presence of Hg^2+^ (**A**). Optimization of concentration of A stand in the presence of Ag^+^ (**B**).

The concentration of the A strand substrate was optimized. At lower concentrations of the A strand, the initial fluorescence value was low, and the change in fluorescence was not obvious. When the concentration of the A strand was higher, the opposite result was observed (Figure 3). It can be seen from Figure 3 that when the concentration of the A strand is higher than 1.5 μM, the change of fluorescence will be decrease, so we chose the optimal A strand concentration of 1.5 μM.

**Figure 4:**
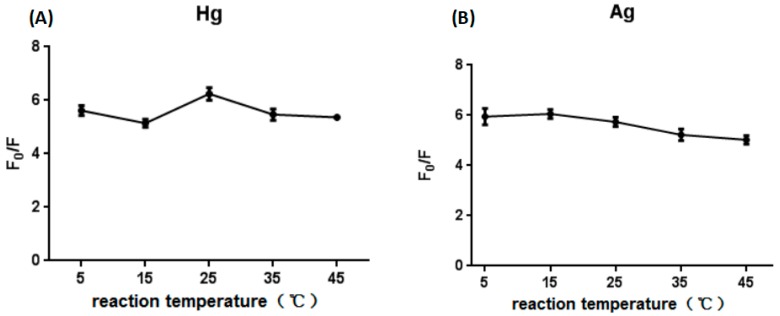
Optimization of the reaction temperature in the presence of Hg^2+^ (**A**). Optimization of the reaction temperature in the presence of Ag^+^ (**B**).

The same as pH value, reaction temperature is an important interference factor in sample detection, so the reaction temperature was optimized. It was found that there was no significant effect on the reaction results when the reaction temperature ranged from 5 °C to 45 °C (Figure 4), which showed that the designed sensor is very stable at different temperatures and suitable for the detection of Hg^2+^ and Ag^+^ in water samples in various seasons. In the laboratory experiment, the reaction results were similar for temperatures of 25 °C and 35 °C. Taking into the actual environmental temperature and the controllability, the optimal reaction temperature is 35 °C.

**Figure 5:**
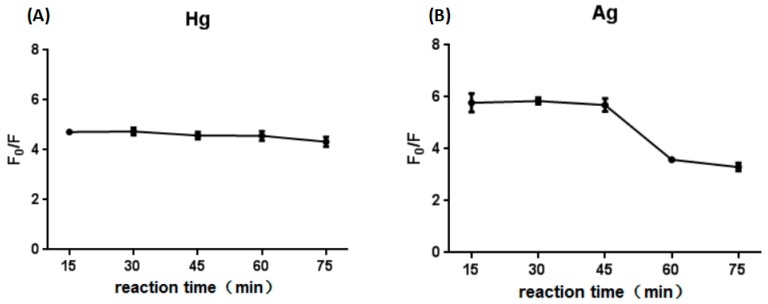
Optimization of the reaction time in the presence of Hg^2+^ (**A**). Optimization of the reaction time in the presence of Ag^+^ (**B**).

The reaction time was optimized. We found that the result of Hg^2+^ was similar for reaction times from 15 min to 75 min, with the result of Ag^+^ was slightly decrease as time increase. In other words, the reaction was so rapid that we could observe the result at 15 min (Figure 5). In addition, after put the experiment results for one day, the fluorescence value increased by 80%, indicating that the experimental results were very stable, and were easier to observe the next day. However, in order to reduce the experimental period, reaction time of 15 min was selected as the optimal condition.

### 4.3. Sensitivity

**Figure 6:**
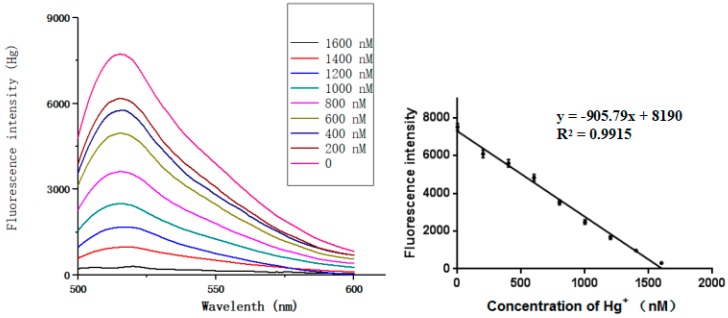
Sensitivity of Hg^2+^ detection.

**Figure 7:**
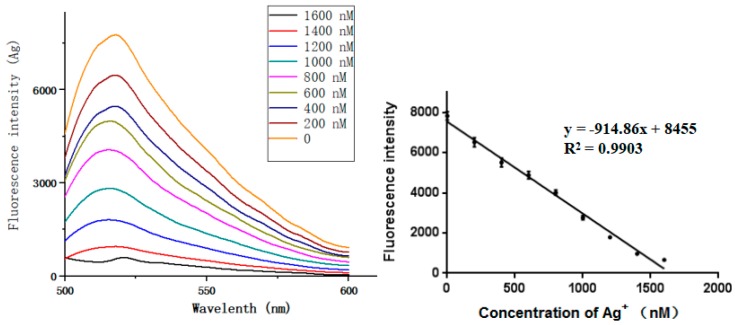
Sensitivity of Ag^+^ detection.

Under optimal conditions, the sensitivity and dynamic range of the sensor were evaluated at different concentrations of the target ions. Experiments on ion target concentration (Hg^2+^ or Ag^+^) were carried out separately. It can be seen that for Hg^2+^ and Ag^+^ concentrations ranging from 0 to 1600 nM, the target concentration and the fluorescence change show a good correlation (Figure 6 and Figure 7). The equation of linear regression for Hg^2+^ was y = −905.79x + 8190 (*R*^2^ = 0.9915), the limit of detection (LOD) based on the 3σ/S calculation (σ is the standard deviation for the blank solution, and *S* is the slope of the calibration curve) was 3.9 pM [3,49,50]. Similarly, the equation of linear regression for Ag^+^ was y = −914.86x + 8455 (*R*^2^ = 0.9903) and the limit of detection was 3.9 pM.

In order to further verify the LOD, the calculated LODs were experimentally verified. Besides the validation of the 3.9 pM ion concentration, the concentrations of 5 pM, 15 pM, 25 pM, 35 pM and 45 pM were also validated, as shown in Table 4. When the ion concentration was less than 15 pM, the recovery rate and SD were high. But when the ion concentration was greater than 15 pM, the recovery rate gradually decreased and the SD gradually stabilized with the increasing of ion concentration. According to the rules, the recovery rate of 100 ± 20% is believed to be credible, so the LOD of the sensor is between 25 pM and 35 pM. For rigorous consideration, the LOD of the method is 35 pM. Although the actual LOD was higher than the calculated LOD, this experiment has a high sensitivity compared with other methods (Table 4).

**Figure 8:**
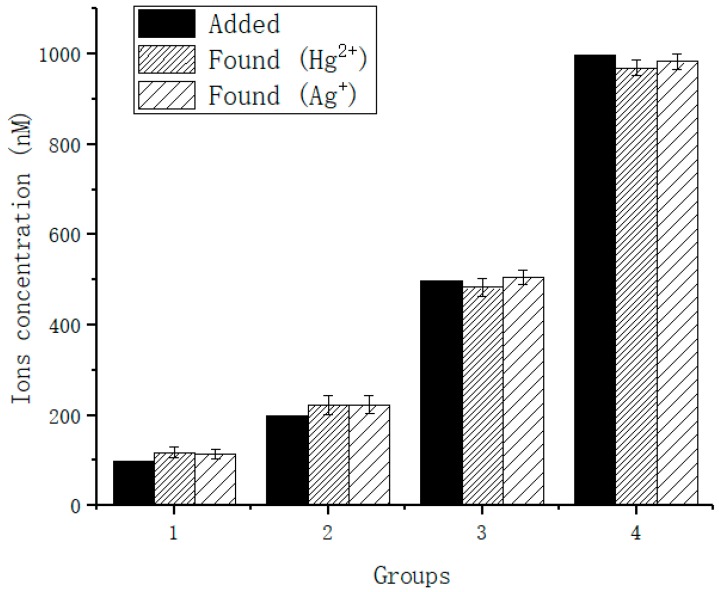
Detection of Hg^2+^ and Ag^+^ in actual samples.

In order to test the practical application of the proposed sensor, the applicability of the sensor to actual samples was investigated. The water samples tested were from rainwater collected from Xi’an, China in July 2018. Hg^2+^ and Ag^+^ were added to the samples to be measured at concentrations of 100 nM, 200 nM, 500 nM and 1 μM. Hg^2+^ and Ag^+^ were measured. As shown below in Figure 8, and the recovery range was 96.6% to 118% (Table 5). These results show that the sensor we designed can be used in actual environmental samples, but the detection error will increase and slightly larger than actual values when the concentration of ions is very low.

**Figure 9:**
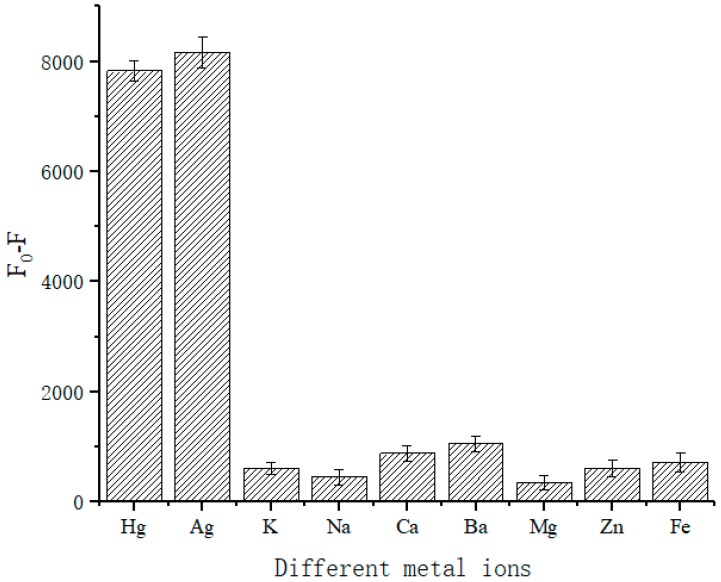
Specific experiment of Hg^2+^ and Ag^+^.

Additionally, we validated the specificity of the designed sensor. The common cations K^+^, Na^+^, Ca^2+^, Ba^2+^, Mg^2+^, Zn^2+^, Fe^3+^ were used as comparisons. The results in Figure 9 show that at each ion concentration of 500 nM, the designed sensor had good specificity for Hg^2+^ and Ag^+^. Conversely, the addition of the other testing species elicited little change in the value of fluorescence.

Finally, the designed sensor was compared with other methods. The colorimetric method often requires less preparation time and reaction time, but it can be seen from Table 6 that the detection limit is higher. Electrochemical methods are highly sensitive, but due to their experimental complexity, the required preparation time is long. The detection limit of the fluorescence method and the time required are intermediate between the colorimetric method and the electrochemical method. The method proposed in this paper is simple, requires only 15 min, and has a low detection limit.

## 5. Conclusions

In summary, we developed a single-molecule multifunctional biosensor based on the ion-mediated strand-displacement reaction, which can not only detect Hg^2+^ and Ag^+^ but also be used to construct an OR logic gate. We optimized the experimental conditions for the sensor and then applied it to actual samples. We demonstrated that the sensor is convenient, fast and can detect steadily at different temperatures. The performance of the sensor in actual samples fully established its feasibility. Because of the difference in the fluorescence intensity of fluorescein under different acid and alkali conditions, the sensor can also be used to preliminarily determine the acid-base balance of water. However, there were also shortcomings in this study, that is, we could determine whether Hg^2+^ and Ag^+^ were present in water samples, but could not specifically distinguish between the two ions. In follow-up studies, we will try to design more sensitive and ingenious sensor platforms.

## Figures and Tables

**Table 1:** DNA strand sequence.

Single Strand	Strand Sequence (5′–3′)
A	FAM–GTACACTGTAAAAAAAAAAAAAAACACTGTG–BHQ

**Table 2:** Levels of each factor.

Factor	Concentration of A Strand (A)	Concentration of Ions (B)	Reaction Time (C)
1	0.5 μM	100 nM	30 min
2	1 μM	200 nM	60 min
3	1.5 μM	500 nM	90 min

**Table 3:** Orthogonal experimental results for Hg^2+^ and Ag^+^.

Experiment Number	Concentration of A Strand (A)		Concentration of Ions (B)		Reaction Time (C)		F_0_–F	
							Hg^2+^	Ag^+^
1	A_1_		B_1_		C_1_		1239.4	*1042.8*
2	A_1_		B_2_		C_3_		1572.9	*1806.4*
3	A_1_		B_3_		C_2_		1535.1	*2010.4*
4	A_2_		B_1_		C_3_		3243.2	*1090.8*
5	A_2_		B_2_		C_2_		6569.5	*4773.2*
6	A_2_		B_3_		C_1_		7675.0	*7103.0*
7	A_3_		B_1_		C_2_		3723.7	*1201.6*
8	A_3_		B_2_		C_1_		8459.9	*9090.4*
9	A_3_		B_3_		C_3_		11,151.3	*10,298.2*
	Hg^2+^	Ag^+^	Hg^2+^	Ag^+^	Hg^2+^	Ag^+^		
K_1_	4347.4	*4859.6*	8206.3	*3335.2*	17,374.3	*17,236.2*		
K_2_	17,487.7	*12,967.0*	16,602.4	*15,670.0*	11,828.4	*7985.2*		
K_3_	23,334.9	*20,590.2*	20,361.4	*19,411.6*	15,967.5	*13,195.4*		
k_1_	1449.1	*1619.9*	2735.4	*1111.7*	5791.4	*5745.4*		
k_2_	5829.2	*4322.3*	5534.1	*5223.3*	3942.8	*2661.7*		
k_3_	7778.3	*6863.4*	6787.1	*6470.5*	5322.5	*4398.5*		
R	6329.2	*5243.5*	4051.7	*5358.8*	1848.6	*3083.7*		

**Table 4:** The detection of low ion concentration.

	Added	Detected	Recovery (%)	SD
Hg^2+^	3.9 pM	10.2 pM	262	4.1
	5.0 pM	13.6 pM	272	3.6
	15.0 pM	23.3 pM	150	3.2
	25.0 pM	30.3 pM	121	4.7
	35.0 pM	38.4 pM	110	4.6
	45.0 pM	47.7 pM	106	5.0
Ag^+^	3.9 pM	12.3 pM	315	5.4
	5.0 pM	15.3 pM	306	2.2
	15.0 pM	21.3 pM	142	3.7
	25.0 pM	31.1 pM	124	3.6
	35.0 pM	39.0 pM	111	5.4
	45.0 pM	48.1 pM	107	4.7

**Table 5:** Detection of Hg^2+^ and Ag^+^ in actual samples.

	Added	Detected	Recovery (%)	SD
Hg^2+^	100 nM	118 nM	118	12.4
	200 nM	222 nM	111	21.8
	500 nM	483 nM	96.6	19.4
	1000 nM	969 nM	96.9	16.7
Ag^+^	100 nM	114 nM	114	11.4
	200 nM	223 nM	115	20.3
	500 nM	505 nM	101	15.4
	1000 nM	983 nM	98.3	17.9

**Table 6:** Comparison of different detection methods.

Detection Method	LOD	Analytical Range (nM)	Reaction and Incubation Time	Ref.
Colorimetric	10 pM	1.0 × 10^−2^–1	45 min	[40]
	1 nM	1–1.0 × 10^3^	6 day	[41]
	2 nM	2–1.0 × 10^2^	3 h	[51]
Electrochemical	0.2 nM	0.5–1.0 × 10^3^	50 min	[52]
	0.12 pM	0.2 × 10^−3^–35	2 h	[38]
	0.1 nM	0.1–1 × 10^4^	5 day	[53]
Fluorometric	30 nM	0–117.0 × 10^6^	1 h	[21]
	9.5 nM	32–1.8 × 10^3^	30 min	[54]
	3 nM	5–1.0 × 10^3^	1 min	[55]
	35 pM	0–1.6 × 10^3^	15 min	This work
